# A systematic review of noninvasive laboratory indices and elastography to predict hepatic decompensation

**DOI:** 10.1097/HC9.0000000000000675

**Published:** 2025-03-24

**Authors:** John Grady, Michael Song, Whitney Townsend, Nadim Mahmud, Elliot B. Tapper, Neehar D. Parikh

**Affiliations:** 1 Department of Internal Medicine, University of Michigan, Ann Arbor, Michigan, USA; 2 Division of Gastroenterology and Hepatology, Department of Internal Medicine, Perelman School of Medicine, University of Pennsylvania, Philadelphia, Pennsylvania, USA; 3 Department of Medicine, Corporal Michael J. Crescenz VA Medical Center, Philadelphia, Pennsylvania, USA; 4 Leonard David Institute of Health Economics, University of Pennsylvania Perelman School of Medicine, Philadelphia, Pennsylvania, USA; 5 Department of Internal Medicine, Division of Gastroenterology and Hepatology, University of Michigan, Ann Arbor, Michigan, USA; 6 Gastroenterology Section, Ann Arbor VA Healthcare System, Ann Arbor, Michigan, USA

**Keywords:** ascites, decompensated cirrhosis, hepatic encephalopathy, variceal bleed

## Abstract

**Background:**

Hepatic decompensation carries profound implications for patient quality of life and risk of mortality. We lack comparative data on how noninvasive tools perform in risk stratification for those with compensated cirrhosis. We performed a systematic review to assess the performance of laboratory and transient elastography–based models for predicting hepatic decompensation in patients with compensated cirrhosis.

**Methods:**

The following databases were searched by an informationist to identify relevant studies, including adult patients with compensated cirrhosis from inception to August 2023: Medline, Embase, Scopus, Web of Science, and ClinicalTrials.gov. Title and abstract screening followed by full-text review were performed by 2 independent reviewers, and data abstraction was completed using standardized forms. Studies of patients with decompensation at baseline (defined by ascites, variceal bleeding, and HE) or any primary hepatic malignancy were excluded. The primary outcome was hepatic decompensation, as defined above. Pooled HRs were calculated using the common-effect inverse-variance model.

**Results:**

Forty-four full-text studies met the inclusion criteria. Across 52,589 patients, the cumulative incidence of any decompensation was 17.9% over a follow-up time of 111,401 patient years. Pooled risk estimates for all-cause decompensation demonstrated that MELD (HR: 1.08; 95% CI: 1.06–1.10), albumin–bilirubin (HR: 2.13, 95% CI: 1.92–2.36), fibrosis-4 (HR: 1.04, 95% CI: 1.03–1.06), albumin–bilirubin–fibrosis-4 (HR: 1.25, 95% CI: 1.18–1.33), and liver stiffness by transient elastography (HR: 1.04; 95% CI: 1.04–1.05) predict decompensation.

**Conclusions:**

Available blood and imaging-based biomarkers can risk-stratify patients for hepatic decompensation. Changes in albumin–bilirubin appear to have the highest discrimination in predicting decompensation events.

## INTRODUCTION

The prevalence of cirrhosis in the United States now exceeds 2 million adults.^1^ The median survival for patients with cirrhosis is 12 years, with a range from 2 to 20 years.^2^ This wide range reflects the need for additional measures to improve the precision of prognoses provided to patients. Perhaps unsurprisingly, patients report difficulty in understanding their prognosis after being given a diagnosis of cirrhosis.^3^ Their future risk of ascites, HE, and variceal bleeding is what determines the patient’s quality of life and risk of death. Thus, accurate prognostic models in both stages of cirrhosis can inform clinical decision-making and provide guidance for patients.

Many tools have been explored for the purpose of prognostication in the setting of cirrhosis. The MELD and the Child–Turcotte–Pugh (CTP) scores accurately predict survival in decompensated patients; however, these indices poorly discriminate survival in compensated cirrhosis and were not intended to predict the risk of decompensation.^4^^,^^5^ Alternatively, invasive measures such as the HVPG can be difficult to obtain in many clinical settings. Noninvasive risk-stratification alternatives have been proposed, but data validating these measures or the comparable ability to predict decompensation are limited.^6^ The emergence of transient elastography (TE) and magnetic resonance elastography (MRE) has prompted some to suggest that liver stiffness measurement (LSM) could be useful as a predictive tool for decompensation.^7^^,^^8^ Beyond these, numerous studies have attempted to evaluate risk scores in compensated disease, either through re-purposing existing noninvasive scores or through creating new models. However, data comparing these tools are also limited, and thus, clinicians lack guidance on which tools to use in clinical practice when evaluating patients with compensated cirrhosis.

We aimed to perform a systematic review to assess the performance of laboratory and elastography-based models for prediction of hepatic decompensation in patients with compensated cirrhosis and to compare estimates of predictive performances.

## METHODS

### Search strategy

The following databases were searched by a health informationist from the time of inception to August 17, 2023, in order to identify relevant articles, trials, or meeting abstracts addressing the prediction of future decompensation in a compensated cirrhosis cohort: PubMed.gov, Elsevier Embase (including Embase Classic), Elsevier Scopus, Web of Science Core Collection (SCI-EXPANDED; SSCI; A&HCI; CPCI-S; CPCI-SSH; BKCI-S; BKCI-SSH; ESCI; CCR-EXPANDED), and ClinicalTrials.gov. Each search utilized controlled vocabulary whenever possible, supplemented with title and abstract keywords. No limits were applied to the search. The complete search strategies are detailed in the supplement. A set of sentinel articles were identified before the search process and were used to generate search terms and test the effectiveness of the strategies in each database. Conference proceedings and clinical trials were included via searches of Embase, Scopus, Web of Science, and ClinicalTrials.gov. Reference tracking was performed on relevant articles to identify additional potentially relevant studies. Original search strategies were developed in Pubmed and translated as appropriate to the other databases using the Systematic Review Accelerator Polyglot tool.^9^ Citations were deduplicated using a modified version of the Bramer Method.^10^ If studies described overlapping cohorts, the most recent study cohort was used. Titles, abstracts, and full texts were sequentially screened against inclusion and exclusion criteria (detailed below) by 2 independent reviewers (John Grady and Michael Song), with discrepancies resolved through discussion and a third reviewer (Neehar D. Parikh) when necessary. The protocol was registered on PROSPERO (CRD42022316507).

### Study selection

Studies of adults (≥18 y of age) with a confirmed diagnosis of cirrhosis via biopsy, labs, and/or imaging that evaluated readily available tests in clinical settings, including laboratory-based indices and elastography-based indices, were included. Exclusion criteria were the following: decompensated cirrhosis, HCC, or any other primary or secondary malignancy of the liver at baseline. Unpublished studies and conference abstracts were also excluded, as were studies that examined proprietary laboratory algorithms or imaging-based tests that are not routinely clinically available. Of note, studies that did not meet inclusion or exclusion criteria overall were included only if they contained extractable estimates of decompensation in patient subgroups that fulfilled inclusion and exclusion criteria.

### Outcome

The primary outcome was incident cirrhotic decompensation, defined by the development of ascites, variceal bleeding, and/or HE. All data were extracted by 2 independent reviewers (John Grady and Michael Song) using standardized forms.

### Statistical analysis

All analyses were conducted using Stata (version 18). In studies where HRs for decompensation as predicted by a clinical tool were reported, pooled HRs were calculated using the common-effect inverse-variance model, in which parameter estimates from individual studies were weighted by the reciprocals of their squared SEs, thereby conveying greater weight to larger studies. Heterogeneity across studies was assessed using *I* ^2^, derived from *Q* (the chi-squared statistic) and its degrees of freedom. *I* ^2^ describes the percentage variability in effect estimates secondary to heterogeneity rather than chance. We attempted to analyze for causes of heterogeneity through subgroup analyses by etiology of liver disease; however, there was not enough granularity of the reported data to do so for all indices, and thus, we were only able to conduct subgroups for MELD and TE.

### Comparison of prognostic systems

As HRs were calculated per unit increase in the incorporated laboratory and imaging models, a direct comparison was limited by differences in the significance of discrete units to their individual models (ie, the disparity between a one-point change in albumin–bilirubin [ALBI] and a one-point change in MELD). Standardization of data was therefore performed by calculating *Z*-scores, by dividing the natural log of the effect size (HR) by the SE of the HR. Pooled HRs were also converted to Cohen’s *d* for standardized estimation of effect size (value=0.2 represents a small effect size; value=0.5 represents a medium effect size; value=0.8 represents a large effect size).

### Quality assessment

Two review authors (John Grady and Michael Song) evaluated the risk of bias in included studies using the Quality In Prognosis Studies (QUIPS) tool.^11^

### Publication bias

Publication bias was assessed using funnel plots of the effect estimate (represented as the natural log of the HR) against the SE. The SE of the effect estimate was selected as the measure of study size and was represented on the vertical axis with a reversed scale in which larger studies were concentrated at the top. A triangle centered on the average effect summary estimate and extending 1.96 SEs on either side was presumed to incorporate 95% of studies if bias was absent and the fixed effect assumption (that the true HR is the same in each study) was valid. The Egger’s test was used to test for funnel plot asymmetry, in which a linear regression was performed of the effect estimates (natural log of the HR) on their SEs, weighting by 1/(variance of the HR).

## RESULTS

### Search results

**FIGURE 1:**
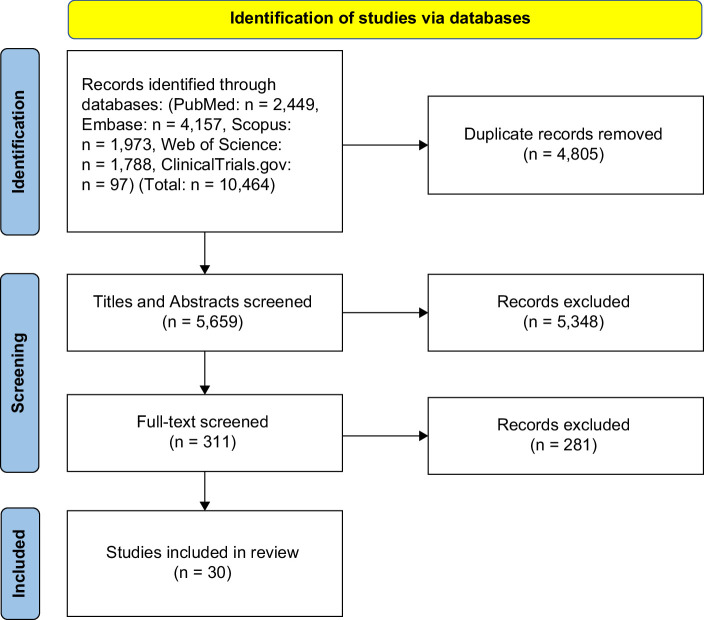
PRISMA flow diagram of study screening and selection.

The initial literature search identified 12,641 citations. After the removal of duplicates, 6879 titles and abstracts were screened. Of these, 377 full-text reports were assessed for eligibility. Forty-four studies ultimately met inclusion criteria (Figure 1).^6^^–^^8^^,^^12^^–^^52^

### Characteristics of included studies

The 44 included studies were published between 1999 and 2023, including a total study population of 52,757 patients (Table 1). A total of 9444 (17.9%) patients developed hepatic decompensation over 111,401 patient-years of follow-up, yielding an incidence rate of 84.8 per 1000 person-years. The most common decompensation event was ascites development (65.7%), followed by variceal bleeding (14.1%) and HE (12.5%); however, there was a lack of granular data on time to individual decompensation events.

**TABLE 1:** Main characteristics of the included studies

Study, country	Study description	Patient population	Years of inclusion	Length of follow-up (mean/median)	N (M/F)	Race/ethnicity	Median/mean age, yr	Etiology of cirrhosis (n)	Total decompensation (n)	Tests evaluated and AUROC (if available)
Hu and Tong 1999^12^, USA	Prospective cohort, single center	HCV	1990–1995	54 mo	112 (56/56)		52.1	HCV (49 treated with IFN)	24 (Ascites 10, HE 5, VB 4)	Platelet count, albumin, INR
Ripoll et al 2007^6^, Spain, USA, UK	Prospective nested cohort within RCT, multicenter		1993–1999	51.1 mo	213 (126/87)		54	MASLD (162), HCV (134), HBV (8), EtOH (51), cryptogenic (10), others (10)	62 (Ascites 46, HE 17, VB 6)	MELD c-statistic 0.64; 95% CI: 0.55–0.72)
Westbrook et al 2011^13^, UK	Prospective cohort, single center	Pregnant patients	1984–2009	Duration of pregnancy	62 (0/62)		29	Autoimmune (27), EtOH (10), viral (6), A1AT (4), Budd–Chiari (2), other (7)	6 (Ascites 2, HE 1, VB 3)	MELD AUROC 0.80 (95% CI: 0.66–0.93)
Berzigotti et al 2011^14^, Italy	Retrospective cohort, single center		2002–2007	28 mo	63 (NR/NR)		NR in subgroup	Multiple etiologies (NR in subgroup)	16 (Ascites 14, HE 0, VB 2)	Bilirubin
Berzigotti et al 2011^15^, US, UK, Spain	Prospective cohort from RCT, multicenter		1993–1999	59 mo	161 (95/66)		54	Viral (109), EtOH (35), cryptogenic (8), other (9)	48 (Ascites 33, HE 15, VB 5)	MELD, albumin
Robic et al 2011^7^, Europe	Prospective cohort,		2005–2006	16.1 mo	65 (36/29)			All etiologies included. Subgroup etiologies NR.	18 (Ascites, HE, and VB NR in cirrhosis subgroup)	LSM by TE AUROC 0.734 [0.609–0.859]
Kim et al 2012^16^, South Korea	Prospective cohort, single center	HBV	2005–2007	42.1 mo	217 (141/76)		50.1	HBV (19.3% treated with antiviral prior to study)	26 (Ascites 22, HE 11, VB 9)	LSM by TE AUROC 0.773 (95% CI: 0.686–0.86)
Procopet et al 2014^17^, Romania	Retrospective cohort, single center		2006–2008	28.1 mo	280 (148/132)		56	HCV (120), HBV (40), EtOH (77), MASLD (23), others (12)	61 (Ascites 54, HE 8, VB 5)	Bilirubin, INR, AST, MELD
Pérez-Latorre et al 2014^18^, Spain	Retrospective cohort, single center	HCV cirrhosis with and without HIV coinfection	2005–2011	42 mo	60 (43/17)		46 (co-infected), 51 (mono-infected)	HCV (txt NR)	8 (Ascites, HE, and VB NR)	LSM by TE AUROC 0.85 (95% CI: 0.69–1.00)
Karagozian et al 2014^19^, USA	Retrospective cohort, academic tertiary care	HCV treated with peg-IFN and ribavirin	2001–2010	55 mo	153 (101/52)	White (122), African American (6), Hispanic (10), Other (15)	57	HCV (26% achieved SVR)	23 (Ascites 13, HE 11, VB 6)	Platelet count
Colecchia et al 2014^20^, Italy	Prospective cohort, academic tertiary care		2010–2012	24 mo	92 (64/28)		56.5	HCV (0% antiviral txt)	30 (Ascites 26, HE 8, VB 4)	MELD
Wang et al 2014^21^, Taiwan	Prospective cohort, academic tertiary care		2008–2011	36.9 mo	220 (135/85)		56.7	HBV (119, 74.8% SVR), HCV (67, 29.9% SVR), HBV + HCV (19), nonviral (15)	9 (Ascites 2, HE 0, VB 6)	TE AUROC 0.929 (95% CI: 0.875–0.984)
Sultanik et al 2016^22^, France	Retrospective cohort, single center		2006–2015	23.5 mo	341 (225/116)		56	HCV (13.2% SVR, 66.3% previous antiviral txt)	56 (Ascites, HE, and VB NR)	TE AUROC 0.70 (95% CI: 0.63–0.76)
Calzadilla-Bertot et al 2016^23^, Cuba	Prospective cohort, tertiary academic care center		2004–2007	46.3 mo	250 (96/154)		60	HCV (0% SVR)	55 (Ascites 28, HE 6, VB 21)	Platelet count, AST/ALT ratio, Child–Pugh score
Takuma et al 2017^24^, Japan	Prospective cohort, single center		2010–2013	44.6 mo (all pts, subgroup NR)	280 (159/129)		67	HCV (166, SVR NR), HBV (45), HBV + HCV (9), EtOH (20), other (40)	35 (Ascites 20, HE 5, VB 10)	MELD c-statistic 0.71 (95% CI: 0.65–0.76). Spleen stiffness by ARFI c-statistic 0.84 (95% CI: 0.79–0.90); AUROC 0.84 (95% CI: 0.76–0.91)
Dillon et al 2018^25^, Ireland	Prospective cohort, multicenter		2010–2015	35.5 mo	244 (77/167)		56.4	Viral (64), EtOH (106), MALSD (41), other (33)	21 (Ascites, HE, and VB NR)	TE
Wu et al 2018^26^, China	Prospective cohort within RCT, multicenter		2013–2015	23.9 mo	405 (310/95)		48	HBV	16 (Ascites 6, HE 2, VB 8)	TE c-statistic 0.76 (95% CI: 0.59–0.93)
Guha et al 2019^27^, UK, Ireland, Egypt	Prospective cohort with a prospective and a retrospective validation cohort, multicenter		Primary cohort initiated 2010. Prospective validation: 2011–2015. Retrospective validation: initiated 2006	55.1 mo	Primary (Nottingham): 145 (95/50)		61	HCV (17), HBV (1), EtOH (63), MASLD (43), cryptogenic (3), other(16)	Ascites 71.4%, HE 7.2%, VB 21.4%	C-statistic: ALBI 0.75 (95% CI: 0.63–0.83). MELD 0.70 (95% CI: 0.58–0.80). ALBI-FIB-4 0.81 (95% CI: 0.72–0.87)
					Prospective (Dublin) Validation: 141 (99/42)		54	HCV (42), HBV (7), EtOH (56), MASLD (12), cryptogenic (3), other (21)	Ascites 29.4%, HE, 23.5%, VB 47.1%	C-statistic: ALBI 0.74 (95% CI: 0.63–0.84). MELD 0.70 (95% CI: 0.60–0.79). ALBI-FIB-4 0.78 (95% CI: 0.68–0.85)
					Prospective (Egypt) Validation: 93 (70/23)		53	HCV (32), HBV (3), EtOH (7), MASLD (44), cryptogenic (6), other (1)	Ascites 57.1%, HE 14.3%, VB 22.9%	
Schwarzer et al 2020^28^, Austria	Retrospective cohort, multicenter		2007–2017, 2010–2014 (2 different centers)	49 mo	194 (108/86)		56	HCV (123), HBV (11), EtOH (19), MASLD (18), Other (23)	35 (Ascites 30, HE 10, VB 5)	MELD AUROC 0.75 (95% CI: NR)
Asesio et al 2022^29^, France	Retrospective cohort, single center		2012–2015	43.2 mo	455 (326/129)		57.7	HCV (193), HBV (46), EtOH (77), MASLD (113), other (26)	37 (Ascites 28, HE 11, VB 9)	TE
Gidener et al 2021^8^, USA	Retrospective cohort, single center	NAFLD	2007–2019	52.8 mo	194 (71/123)	White (186), all other races (8)	64	MASLD	68 (Ascites 30, HE 20, VB 2)	MRE
Wu et al 2021^30^, China	Prospective combined cohorts from RCT and observational study	HBV receiving antiviral therapy	2012–2015	54 mo	937 (695/242)		47.1	HBV (64.9% entecavir monotherapy, 26.9% entecavir + thymosin-alpha1, 8.2% lamivudine + adefovir)	40 (Ascites 24, HE 1 VB 20)	TE
Hsu et al 2021^31^, USA	Retrospective cohort, sourced from national database		2001–2015	17.2 mo	Nonviral: 1978 (967/1011)		60	EtOH (561), MASLD (760), PBC (422), PSC (109), AIH (118), other (92)	309 (Ascites 146, HE 122, VB 41) at year 1	AUROC at 1 y: MELD 0.69 (95% CI: 0.688–0.693). ALBI 0.708 (95% CI: 0.704–0.712). ALBI-FIB-4 0.734 (95% CI: 0.73–0.738)
					Viral: 1744 (1076/668)		57	HCV (1639), HBV (321), EtOH (424), PBC (133), PSC (46), AIH (54), other (53)	330 (Ascites 153, HE 107, VB 70)	AUROC at 1 y: MELD 0.656 (95% CI: 0.653–0.66). ALBI 0.728 (95% CI: 0.725–0.731). ALBI-FIB-4 0.741 (95% CI: 0.737–0.745)
Costa et al 2021^32^, Austria	Prospective cohort, academic tertiary care center		2017–2019	12.2 mo	78 (52/26)		56.8	Viral (27), EtOH (16), EtOH + viral (7), MASLD (15), other (12)	17 (Ascites, HE, and VB NR)	IL-6
Calzadilla-Bertot et al 2021^33^, Spain, Australia, Hong Kong, Cuba	Retrospective cohort, multicenter	NAFLD	1995–2013	61.2 mo	299 (139/160)	White (181), Hispanic (66), Asian (48), African American (4)	56.8	MASLD	81 (Ascites 60, HE 5, VB 16)	Derivation cohort AUROC at 5 y: MELD 0.69 (95% CI: 0.66–0.75). ALBI 0.72 (95% CI: 0.62–0.84). ABIDE 0.80 (95% CI: 0.73–0.84)
Morisco et al 2021^34^, Italy	Prospective cohort, multicenter	HCV treated with DAAs	2015–2017	28.4 mo	706 (387/319)		64.1	HCV (97.3% with SVR)	28 (Ascites 16, HE 5, VB 15)	TE
Lee et al 2021^35^, South Korea, Hong Kong	Post hoc analysis of 2 prospective cohorts, multicenter	HBV receiving antiviral txt	2006–2008, 2006–2012	58.1 mo	818 (519/299)		54.9	HBV (entecavir 54.9%, tenofovir 44.4%, other 0.7%)	21 (Ascites 15, HE 8, VB 5)	TE, ALT, platelet count, bilirubin, albumin, INR, eGFR
Mahmud et al 2022^41^, USA	Retrospective cohort	Veterans with cirrhosis undergoing surgery	2008–2015	3 mo	4712 (4580/132)	White (2981), Black (741), Hispanic (323), Asian (51), Other (616)	64	HCV (612), HBV (73), EtOH (1662), HCV + EtOH (1388), MASLD (585), other (392)	408 (Ascites 199, HE 121, VB 72)	MELD AUROC at 90 d post-op— Derivation: 0.581 (0.515–0.647); Validation: 0.646 (0.546–0.746)
Franzè et al 2022^42^, Italy	Retrospective cohort, single center	HCV starting direct acting antiviral drugs	2015–2016	63 mo	324 (172/152)		67	HCV	24 (Ascites NR, HE NR, VB NR)	TE
Fujiwara et al 2022^40^, USA	Prospective, single center		2004–2006	66 mo	122 (80/42)		51	HCV (48), HBV (10), EtOH (17), MASLD (10), cryptogenic (14), other (24)	29 (Ascites 13, HE, 16, VB 3)	C-statistic MELD 0.54 (95% CI: 0.50–0.64). ALBI-FIB-4 0.61 (0.54–0.70).
Gidener et al 2022^36^, USA	Retrospective cohort, multicenter		2007–2009	122.5 mo	277 (157/120)	White (231), African American (15), Other (7), Asian (5), Missing (19)	57	HCV (103), HBV (7), EtOH (18), MASLD (63), other (86)	83 (Ascites 78, HE 54, VB 21)	LSM by MRE c-statistic 0.67 (95% CI: NR). MELD c-statistic 0.66 (95% CI: NR).
Jachs et al 2022^37^, Austria	Retrospective cohort, unspecified location		2007–2020		30 (NR/NR)		NR	HBV (undergoing txt with nucleotide analog)	5 (Ascites, HE, and VB NR)	AUROC at 3 y: LSM by TE 0.94 (95% CI: 0.87–1.00). MELD 0.87 (95% CI: 0.73–1.00)
Liu et al 2022^43^, Asia, Egypt	Retrospective cohort, multicenter		2009–2021	50.1 mo (derivation cohort); 29.8 mo (validation cohort)	Derivation: 197 (95/102). Validation: 770 (561/209)		Derivation: 65. Validation: 53	Viral (D: 73, V: 641), EtOH (D: 26, V: 28), MASLD (D: 65, V: 45), other (D: 33, V: 56)	Derivation: 53 (Ascites, HE, and VB NR). Validation: NR	AUROC at 3 y: MELD 0.68 (95% CI: 0.55–0.80). ALBI 0.71 (95% CI: 0.59–0.83). ALBI-FIB-4 0.75 (95% CI: 0.63–0.86). LSM >20 kPa 0.62 (95% CI: 0.52–0.73)
Schneider et al 2022^38^, USA, UK	Retrospective cohort, multicenter		1996–2021	36 mo	Explorys training cohort: 6049 (3300/2748)	White (4528), African American (746), Other (151), Asian (40), Hispanic (38), Missing (546)	61	EtOH (576), other (4202), missing (1271)	1510 (Ascites 860, HE 355, VB 143)	MELD, ALBI, EPOD
					Explorys validation cohort: 17,662 (10,246/7416)	White (13,391), African American (2193), Other (547), Asian (203), Hispanic (74)	61	EtOH (2373), other (12,539), missing (2750)	4,286 (Ascites 3119, HE 31, VB 400)	AUROC at 3 y: MELD 0.635 (95% CI: 0.626–0.645). EPOD 0.694 (95% CI: 0.686–0.704). ALBI 0.67 (95% CI: 0.66–0.68)
					PMBB: 1326 (919/406)	White (815), African American (405), Hispanic (47), Other (25), Asian (18), Missing (16)	66.2	EtOH (214), HBV (600), HCV (61), Other (451)	496 (Ascites 198, HE 43, VB 167)	AUROC at 3 y: MELD 0.55 (95% CI: 0.48–0.63). EPOD 0.692 (95% CI: 0.625–0.759). ALBI 0.67 (95% CI: 0.60–0.73)
					UKB: 317 (220/97)	White (288), Asian (13), African American (5), Other (9), Missing (3)	59	EtOH (170), HBV (39), HCV (1), Other (117)	75 (Ascites 52, HE 0, VB 15)	AUROC at 3 y: EPOD 0.77 (95% CI: 0.69–0.85). ALBI 0.73 (0.63–0.83)
Jindal et al 2022^39^, India	Retrospective cohort, single center		2010–2019	26 mo	626 (465/161)		50.8	HCV (104), HBV (64), EtOH (190), MASLD (165), Other (103)	132 (Ascites 105, HE 39, VB 40)	LSM by TE >22 kPa AUROC at 1 y: 0.723±0.028
Wong et al 2022^44^, Singapore and China	Retrospective cohort, multicenter		2013–2017	39 mo	633 (437/196)		53	HCV (247), HBV (251), EtOH (20), MALSD (39), Other (76)	60 (Ascites 51, HE 12, VB 19)	MELD AUROC at 1 y: 0.74 ( 95% CI: 0.60–0.89), 3 y: 0.63 (0.54–0.72), and 5-year: 0.61 (0.51–0.72). ALBI AUROC at 1 y: 0.79 (0.66–0.92), 3-year: 0.66 (0.57–0.74), and 5-year. ALBI-FIB-4 AUROC at 1 y: 0.83 (0.71–0.94), 3-year: 0.73 (0.65–0.81), and 5-year: 0.70 (0.60–0.80). LSM ≥20 kPa—tAUC at 1 y: 0.62 (0.51–0.74), 3-year: 0.65 (0.58–0.72), and 5-year: 0.52 (0.40–0.63)
Yu et al 2022^45^, China	Retrospective cohort, multicenter		2016–2020	37.6 mo	689 (441/248)		54	Viral (485), EtOH (32), Cholestatic and autoimmune (91), Other (81)	184 (Ascites 113, HE 14, VB 57)	C-index MELD training cohort 0.5 (0.41–0.59); Validation: 0.62 (0.56–0.68). ALBI training cohort: 0.61 (0.52–0.7); Validation: 0.68 (0.62–0.73). FIB-4 training cohort: 0.73 (0.66–0.8); Validation: 0.68 (0.63–0.74). ALBI-FIB-4. Training cohort: 0.7 (0.62–0.78); Validation: 0.7 (0.65–0.75)
Karagiannakis et al 2023^46^, Greece	Retrospective cohort		2018–2021	31 mo	74 (NR/NR)		56	NR for compensated cohort	30 (Ascites NR, HE NR, VB NR)	Spleen stiffness
Kim et al 2023^47^, South Korea	Retrospective cohort, single center	HBV cirrhosis	2006–2014	93 mo	482 (320/162)		54	HBV	48 (Ascites 45, HE 26, VB 25)	AUROC ALBI: 1 y 0.8153 (0.7111–0.9196); 3 y 0.7451 (0.6425– 0.8476); 5 y 0.7541 (0.6705–0.8376). ALBI-FIB-4: 1 y 0.8168 (0.7109–0.9227); 3 y 0.7730 (0.6791–0.8669); 5 y 0.7743 (0.6960–0.8526)
Loomba et al 2023^48^, Europe, North America, South America, Asia	Retrospective analysis of 4 large, randomized placebo-controlled trials	This study used data from RCTs of selonsertib and simtuzumab in participants with MASLD	2013–2014, 2017–2018	16.2 mo	734 (273/461)		59	MASLD	23 (Ascites 15, HE 5, VB 3)	TE
Marie et al 2023^49^, Egypt	Prospective cohort	HCV cirrhosis patients undergoing DAA therapy	2016–2018	24 mo	1789 (878/911)		54.85	HCV (100% SVR)	184 (Ascites 197, HE 7, VB 15)	Before DAA therapy: ALBI AUROC 0.609. After DAA ALBI AUROC AUC 0.597
Semmler et al 2023^50^, Austria	Retrospective cohort, single center		2007–2020	76 mo	757 (473/284)		52	HCV (384), HBV (48), EtOH (54), MASLD (110), cryptogenic (4), other (157)	83 (Ascites NR, HE NR, VB NR)	LSM AUROC at 1 y: 0.813 [95% CI: 0.749–0.876]
Shearer et al 2023^51^, UK	Retrospective cohort, multicenter		2008–2019	37.2 mo	3028 (1766/1262)	White (1918), Asian (335), Black (76), Other (672), Mixed (27)		Viral (292), EtOH (333), AMS, autoimmune (210), metabolic (71), missing (1287)	157	TE
Wong et al 2023^52^, China, India, Singapore, Italy	Retrospective cohort, multicenter		2014–2017	40 mo	1159 (776/383)	Caucasian (357), Chinese (328), Indian (310), Malay (111), Arabic (28), Others (25)	55	HCV (650—all SVR), HBV (247), EtOH (105), MASLD (102), other (55)	83 (Ascites 73, HE 15, VB 15)	TE

Abbreviations: A1AT, alpha-1 antitrypsin; AIH, autoimmune hepatitis; ALBI, albumin–bilirubin; ARFI, acoustic radiation force impulse; DAA, direct-acting antiviral; FIB-4, fibrosis-4; IFN, interferon; INR, international normalized ratio; LSM, liver stiffness measurement; MASLD, metabolic dysfunction–associated steatotic liver disease; MRE, magnetic resonance elastography; NR, not reached; PBC, primary biliary cholangitis; PMBB, Penn Medicine BioBank; ﻿PSC, primary sclerosing cholangitis; RCT, randomized control trial; SVR, sustained virological response; TE, transient elastography; UKB, United Kingdom Biobank; ﻿VB, variceal bleed.

The mean age of participants was 57.9 years (SD 2.3 y). Across the 41 studies that reported sex, 62% were males and 38% were females. Eight studies reported race or ethnicity (Table 1). Twenty-six studies evaluated multiple etiologies of cirrhosis, 9 included only HCV cirrhosis, 6 evaluated only HBV cirrhosis, and the remaining 3 studies only assessed metabolic dysfunction–associated steatotic liver disease cirrhosis.

The quality assessment results are summarized in Supplemental Table S1, http://links.lww.com/HC9/B938. Of 44 studies in total, 26 (59.1%) studies were rated as a low overall risk of bias, 10 (22.7%) were assigned a moderate risk of bias, and 8 (18.2%) were rated as high risk of bias.

### Prediction of all-cause decompensation

Pooled HRs for all-cause decompensation are summarized in Table 2. All HRs represent a single unit change in the corresponding model. AUROC and c-statistics are reported in Table 1.

**TABLE 2:** Pooled HRs, *Z*-scores, and effect size of the included laboratory and imaging models

						Heterogeneity	
Model	Number of studies	References	Pooled risk estimates for all-cause decompensation (per unit)	*Z*-score	Cohen’s *d*	*I* ^2^	*p*
MELD	13	^6^^,^^14^^,^^20^^,^^24^^,^^25^^,^^27^^,^^28^^,^^30^^–^^32^^,^^36^^,^^37^^,^^46^	1.08 (1.06–1.10)	8.145	0.042	46.0%	0.035
ALBI	3	^27^^,^^31^^,^^51^	2.13 (1.92–2.36)	14.365	0.417	19.5%	0.289
ALBI-FIB-4	2	^27^^,^^31^	1.25 (1.18–1.33)	7.310	0.123	94.0%	0.000
FIB-4	4	^27^^,^^31^^,^^36^^,^^51^	1.04 (1.03–1.06)	5.355	0.022	87.0%	0.000
LSM by TE	11	^16^^,^^22^^,^^25^^,^^26^^,^^29^^,^^30^^,^^37^^,^^42^^–^^44^^,^^46^	1.04 (1.03–1.05)	7.994	0.022	69.7%	0.000

*Z*-score=test of significance for weighted average effect size (ln(HR)/SE_ln(HR)_), Cohen’s *d*=standardized mean difference, *I* ^2^=percentage of variation across studies attributable to heterogeneity rather than chance.

Abbreviations: ALBI, albumin–bilirubin; FIB-4, fibrosis-4; LSM, liver stiffness measurement; TE, transient elastography.

### MELD

**FIGURE 2:**
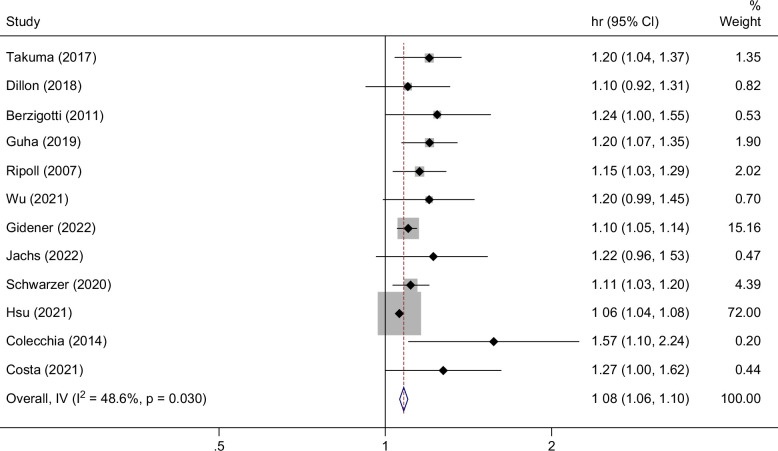
Forest plot displaying the individual and pooled HRs for the risk of hepatic decompensation per unit increase in MELD.

Twenty studies evaluated the MELD as a predictor of future decompensation.^6^^,^^13^^,^^14^^,^^17^^,^^20^^,^^24^^,^^25^^,^^27^^,^^28^^,^^30^^–^^32^^,^^36^^,^^37^^,^^40^^,^^41^^,^^43^^–^^46^ The median baseline MELD was 8.8. Seventeen of these studies assessed varying etiologies of cirrhosis, 2 only investigated HBV cirrhosis, and 1 study evaluated HCV cirrhosis. Thirteen papers reported HRs for MELD, resulting in a pooled HR of 1.08 (95% CI: 1.06–1.10) for the prediction of decompensation (Figure 2).

Stratification of MELD by nonviral (HR: 1.07, 95% CI: 1.05–1.09) and viral (HR: 1.09, 95% CI: 1.06–1.12) etiologies of cirrhosis showed no significant difference in prediction of decompensation (Supplemental Figure S1, http://links.lww.com/HC9/B938). MELD performed similarly in studies with a low risk of bias (HR: 1.07, 95% CI: 1.05–1.09) compared to studies with moderate or high risk of bias (HR: 1.11, 95% CI: 1.07–1.15).

Three studies identified optimal cutoffs for the MELD’s prediction of decompensation. Ripoll et al^6^ reported an optimal cutoff of 10 with a negative predictive value (NPV) of 78%. This compares to an optimal cutoff of 9.5 found by Schwarzer et al.^28^ In pregnant patients with cirrhosis, a MELD score ≥10 yielded a sensitivity of 83% and specificity of 79% for predicting hepatic decompensation during pregnancy.^13^

### Albumin–bilirubin, albumin–bilirubin-fibrosis-4, and fibrosis-4

**FIGURE 3:**
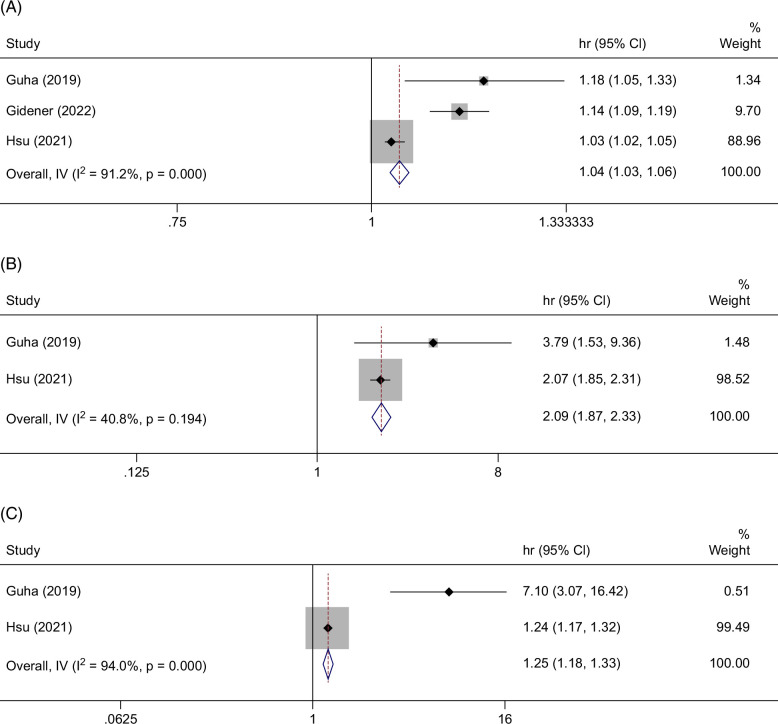
Forest plot showing the individual and pooled HRs for risk of hepatic decompensation per unit increase in (A) fibrosis-4, (B) albumin–bilirubin, and (C) albumin–bilirubin-fibrosis-4.

Four studies were included in the pooled analysis of the fibrosis-4 (FIB-4) score (HR: 1.04, 95% CI: 1.03–1.06) in prognostication of decompensation (Figure 3A).^27^^,^^31^^,^^36^^,^^51^ Three studies met inclusion criteria for pooled analysis of the ALBI (HR: 2.13, 95% CI: 1.92–2.36) and 2 studies for combined ALBI-FIB-4 (HR: 1.25, 95% CI: 1.18–1.33) scores (Figure 3B, C).^27^^,^^31^^,^^51^ The ALBI-FIB-4 outperformed the ALBI in the 5 studies that compared these 2 noninvasive scores by AUROC or c-statistic (Table 1).^27^^,^^31^^,^^43^^–^^45^

### Transient elastography

**FIGURE 4:**
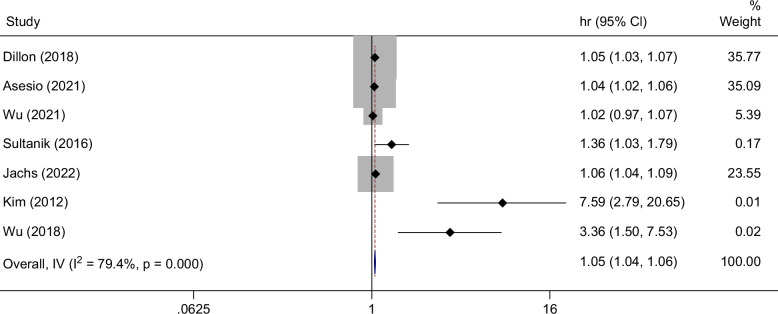
Forest plot displaying the individual and pooled HRs for risk of hepatic decompensation per unit increase (kPa) in liver stiffness measurement by transient elastography.

Nineteen studies evaluated TE in the prediction of hepatic decompensation.^7^^,^^16^^,^^18^^,^^21^^,^^22^^,^^25^^,^^26^^,^^29^^,^^30^^,^^34^^,^^37^^,^^39^^,^^42^^–^^44^^,^^46^^,^^48^^,^^50^^,^^52^ The median baseline LSM was 21.6 kPa. Nine studies assessed viral etiologies of cirrhosis while 10 studies evaluated multiple etiologies. Eleven studies reported HRs (representing a single unit change in kPa) for TE, yielding a pooled HR of 1.04 (95% CI: 1.03–1.05) (Figure 4).

In subgroup analysis, TE performed similarly in decompensation prediction of nonviral (HR: 1.04, 95% CI: 1.03–1.06) and viral (HR: 1.05, 95% CI: 1.04–1.07) etiologies of cirrhosis (Supplemental Figure S2, http://links.lww.com/HC9/B938). Stratification of TE by low risk of bias (HR: 1.04, 95% CI: 1.03–1.05) and moderate or high risk of bias (HR: 1.05, 95% CI: 1.03–1.07) yielded similar results.

Studies also reported on varying dichotomous LSM cutoffs for predicting decompensation. In a study population incorporating various etiologies of cirrhosis, Dillon et al^25^ found no decompensation events in patients with a baseline LSM <21 kPa. Wang et al^21^ reported that at a cutoff of 21.1 kPa, LSM by TE has an AUROC 0.929 (0.875–0.984) and an NPV 99% for hepatic decompensation over a median follow-up of 36.9 months in a cohort of viral causes of cirrhosis. This is consistent with an earlier study of multiple etiologies of cirrhosis, which found that an LSM cutoff of 21.1 kPa had a 100% NPV.^7^ For multiple etiologies of cirrhosis, a cutoff of 22 kPa yielded an NPV of 95% and AUROC of 0.723 for decompensation at 1 year.^39^ Asesio et al^29^ reported HRs of 0.46 (95% CI: 0.23–0.91) and 0.37 (95% CI: 0.18–0.75) in multivariable analysis for favorable Baveno VI (LSM <20 kPa and platelet count >150,000/mm^3^) and Expanded-Baveno VI (LSM<25 kPa and platelet count >110,000/mm^3^) status, respectively. A 2014 study by Pérez-Latorre et al^18^ described an NPV of 97% in patients with a TE value <25 kPa for a composite outcome of liver-related events (first occurrence of decompensation or HCC). Jachs et al^37^ found that a cutoff of <25 kPa had an NPV of 100% for decompensation.

### Magnetic resonance elastography

Two studies assessed LSM by MRE. Gidener et al^36^ reported an HR of 1.26 (95% CI: 1.16–1.36) for cirrhosis decompensation with a c-statistic of 0.67 in 349 patients with cirrhosis of varying etiologies. In a study of 194 NAFLD patients with compensated cirrhosis, Gidener et al found an HR of 1.32 (95% CI: 1.13–1.56) and c-statistic of 0.70 for LSM by MRE for a composite outcome of decompensation or death.

### Other

CTP scores A5, A6, and B7 had a pooled HR of 1.41 (95% CI: 1.33–1.49) across 5 studies (Supplemental Figure S3, http://links.lww.com/HC9/B938).^6^^,^^23^^,^^24^^,^^31^^,^^36^ Seven studies evaluated albumin (HR: 0.89, 95% CI: 0.86–0.92),^6^^,^^15^^,^^28^^–^^30^^,^^37^^,^^43^ 6 studies evaluated total bilirubin (HR: 1.07, 95% CI: 1.05–1.10),^6^^,^^14^^,^^21^^,^^23^^,^^29^^,^^49^ 7 studies evaluated platelet count (HR: 0.99, 95% CI: 0.98–0.99),^6^^,^^12^^,^^23^^,^^30^^,^^37^^,^^42^^,^^43^ 4 studies evaluated INR (HR: 1.09, 95% CI: 0.86–1.39),^6^^,^^23^^,^^42^^,^^49^ and 2 studies evaluated the AST/ALT ratio (HR: 2.01, 95% CI: 1.44–2.80).^23^^,^^33^ Pooled HRs of individual laboratory markers are summarized in Supplemental Table S2, http://links.lww.com/HC9/B938. The APRI, Lok Index, EPOD, and ABIDE scores were evaluated in 1 study each.^31^^,^^33^^,^^38^

In a study including 393 patients with cirrhosis, spleen stiffness by acoustic radiation force impulse had a C-index of 0.843 in univariate analysis with an optimal cutoff of 3.25 m/s resulting in sensitivity, specificity, NPV, and positive predictive value of 94.3%, 65.3%, 98.8%, and 28.0% respectively.^24^ Colecchia et al^20^ reported an OR of 1.11 (95% CI: 1.05–1.17) on multivariable analysis for spleen stiffness by TE for prediction of decompensation. Spleen stiffness had an AUROC 0.710 (*p*=0.003) in a cohort of 74 compensated cirrhosis patients followed for an average of 31 (SD: 18) months.^46^

### Comparison between models

ALBI (*Z*=14.365) outperformed MELD (*Z*=8.145), LSM by TE (*Z*=7.994), ALBI-FIB-4 (*Z*=7.310), and FIB-4 (*Z*=5.355) by *Z*-score. Pooled HRs were also converted to Cohen’s *d* for a simplified interpretation of effect size, with traditional cutoffs of 0.2, 0.5, and 0.8 corresponding to small, medium, and large effect sizes, respectively. All incorporated models demonstrated a small to moderate effect size by Cohen’s *d* with single unit changes, with values of *d*=0.417 for ALBI*, d*=0.042 for MELD, *d*=0.022 for LSM by TE, *d*=0.123 for ALBI-FIB-4, *d*=0.022 for FIB-4, and Table 2.

### Heterogeneity and publication bias

ALBI had *I* ^2^ of 19.5% (*p*=0.289). Moderate heterogeneity was observed in the pooled analyses of MELD (*I* ^2^=46.0%, *p*=0.035), while considerable heterogeneity was seen for FIB-4 (*I* ^2^=87.0%, *p*<0.01), ALBI-FIB-4 (*I* ^2^=94.0%, *p*<0.01), and TE (*I* ^2^=69.7%, *p*<0.01) (Table 2). Funnel plot asymmetry and Eggers test suggested possible publication bias for the prediction of decompensation in pooled analyses of MELD and TE (*p*<0.05).

## DISCUSSION

Multiple laboratory and imaging-based indices have been evaluated for the purpose of risk prediction in cirrhosis. In this systematic review and meta-analysis, we show the predictive capability of the indices for the development of hepatic decompensation and provide a direct comparison of the indices. While MELD was the most widely studied, the ALBI outperformed all other models per unit increase.

### Albumin–bilirubin is the strongest predictor of decompensation

ALBI combines 2 liver function tests in a continuous variable to provide a score that is tailored for the prediction of short-to-medium term liver-related events. Originally derived for risk prediction among patients with HCC undergoing therapy, it was therefore calibrated largely in patients without baseline decompensation.^53^ We confirm its ability to predict decompensation among patients without HCC, with a consistent effect following standardization of the scores. Our pooled HR analysis included only 3 studies examining ALBI; however, one included 2 large cohorts of >1700 patients. The addition of FIB-4, as seen in the ALBI-FIB-4 score, did not provide additional prognostic benefit in the meta-analysis.

### Fibrosis-4 is weakly predictive of decompensation

The FIB-4 alone was the least predictive of decompensation of all models. The performance of FIB-4 may be optimized when combined with additional prognostic markers. The addition of albumin to create the FIB-4+ score is predictive of clinically significant portal hypertension in patients with metabolic dysfunction–associated steatotic liver disease.^54^ In a recent meta-analysis, the combination of FIB-4 and MRE into the MEFIB index yielded a HR of 20.6 (95% CI: 10.4–40.8, *p*<0.001) for the prediction of decompensation.^55^ This synergistic association was seen for a positive cutoff of MRE ≥3.3 kPa and FIB-4 ≥1.6. However, FIB-4 does not optimize ALBI; ALBI-FIB-4 was less predictive than ALBI alone (Table 2).

### Model for end-stage liver disease and Child–Turcotte–Pugh

The MELD serves as a convenient prognostic model given its widespread role in the survival prediction of decompensated cirrhosis and liver transplant allocation. Our meta-analysis showed a predictive power of MELD of 1.08 (95% CI: 1.06–1.10) for hepatic decompensation (*Z*=8.145). MELD was conditioned for risk prediction in patients with decompensated cirrhosis, and for that reason, it is unsurprising that the range of MELD scores in compensated cirrhosis is more restricted, and its predictive ability is limited. The addition of albumin to the MELD 3.0 score may provide improved discriminative ability in compensated patients. Similar to MELD, the prognostic ability of the CTP score is even more limited in compensated cirrhosis, as these patients lack ascites or HE. Further, while the CTP score includes albumin and bilirubin, these variables are weighted in an additive manner compared to the logarithmic and multiplicative weight given to these variables in the ALBI.

### Vibration-Controlled Transient Elastography

TE, like MELD, showed a small effect size in the prediction of decompensation per unit increase by Cohen’s *d*. In evaluating the ideal LSM cutoff for predicting decompensation of cirrhosis by TE, multiple studies reported a strong NPV at a cutoff of 25 kPa. This aligns with the cutoffs proposed by the Baveno VII consensus for ruling in clinically significant portal hypertension, a well-established invasive marker for decompensation risk.^56^ This is a higher threshold compared to findings by Robic and colleagues, who found that a cutoff of 21.1 kPa had an NPV of 100% in varying etiologies of cirrhosis. However, the optimal cutoff point for predicting decompensation may depend on the etiology of cirrhosis, as previous studies have shown a single cutoff of LSM ≥ 25 kPa in obese patients with metabolic dysfunction–associated steatotic liver disease cirrhosis to be a poor predictor of clinically significant portal hypertension.^54^

### Contextual factors

Our systematic review has several notable strengths and limitations. Funnel plot asymmetry and Egger’s test suggested publication bias for MELD and TE in their prediction of hepatic decompensation. Notably, given the limited studies evaluating the other indices, publication bias may be difficult to accurately assess. Aside from the performance of the test itself, publication bias, and true heterogeneity, alternative explanations for these findings could be attributable to the exclusion of abstracts and unpublished data in our systematic review. Our meta-analysis was also limited by the varied statistical methods employed in the individual studies; only studies reporting HRs were represented in the meta-analysis. We observed moderate to considerable heterogeneity for all models, and this trend persisted in the stratified analysis of MELD and TE. This may be attributable to methodological diversity among studies, including variable lengths of follow-up time, varying cohort characteristics depending on underlying liver disease etiology, and differential standards of medical care depending on the temporal and geographic locations of the cohort. Furthermore, 18/44 studies had a moderate or high overall risk of bias; however, stratified analysis by the risk of bias did not reveal differences in decompensation prediction for MELD or TE. The use of composite outcomes was widespread among these 18 studies, necessitating subgroup analysis of small sample sizes to assess the outcome of decompensation in isolation. Patients with cirrhosis are subject to several competing risks and endpoints, including development of HCC, liver transplant, and death, which were not adequately accounted for in several of the included studies. Over half of the studies in our review were retrospective in nature, which raises the possibility of unmeasured confounders impacting the results of our analysis. Further, a large proportion of the study population includes patients with viral hepatitis. However, over the course of the included study period (1999–2023), treatment paradigms for viral hepatitis have dramatically improved, considerably impacting the risk of decompensation. The included studies provided varying details regarding the treatment status—and treatment modalities— of patients (Table 1), confounding the interpretation of pooled results. Similarly, the degree of alcohol consumption in patients with alcohol-associated cirrhosis was not accounted for in studies and serves as another potential confounding variable of this meta-analysis. Further studies with prospective design evaluating risk in compensated cirrhosis, such as the National Institute of Diabetes and Digestive and Kidney Disease sponsored Liver Cirrhosis Network study, may be better equipped to evaluate the noninvasive prediction of decompensation in cirrhosis.^57^

This study is generalizable to patients with cirrhosis, as we excluded chronic liver disease patients without cirrhosis. It is also important to note that our study only reviewed models based on readily available laboratory tests and imaging tests. Predictive models are of greatest use when they impact disease management and can be implemented practically, particularly when used for common disease states like compensated cirrhosis. For instance, scores such as the ALBI can be easily calculated from routine bloodwork prior to visits, allowing for more tailored management decisions during appointments. Identification of compensated cirrhosis patients at risk of decompensation may capture patients who would benefit from non-selective beta blocker initiation. Additionally, patients at high risk of decompensation may benefit from proactive nutritional and frailty assessments rather than reactive management after decompensation. Future studies should focus on noninvasive, cost-effective, and easily accessible predictive models and directly compare these indices. Future studies should assess whether the combination of laboratory and elastography indices has a better discriminant ability to predict decompensation, as this may be a way to improve existing models. Evaluation of noninvasive tests in prospective studies of compensated cirrhosis patients with a clearly defined endpoint of hepatic decompensation is required before recommending the widespread adoption of noninvasive tests to predict decompensation.

In conclusion, this systematic review shows that blood and imaging-based biomarkers can risk-stratify patients for decompensation in varying etiologies of compensated cirrhosis. There was significant heterogeneity in the studies; thus, accurate clinically meaningful cutoffs for the risk of decompensation require further definition; however, in the meantime, changes in ALBI appear to have the best discrimination in predicting decompensation in compensated cirrhosis.

## Supplementary Material
