# Supplementary material for: A systematic review of noninvasive laboratory indices and elastography to predict hepatic decompensation
Source: Hepatol Commun. 2025 Mar 24;9(4):e0675. doi: 10.1097/HC9.0000000000000675 (PMC11936601; doi:10.1097/HC9.0000000000000675)
Supplement: Supplementary file 1 [file hc9-9-e0675-s001.docx]

Supplementary Methods

Search Strategy:

Due to a high volume of retrieval and a high level of irrelevant results, the search was weighted more heavily towards specificity when selecting search terms, with the understanding that the review team will conduct extensive forward and backward citation tracking. Conference proceedings and clinical trials were included via searches of Embase, Scopus, and Web of Science and ClinicalTtrials.gov. Test citations (all identified) were, in Pubmed syntax: 22239518[uid] OR 32978796[uid] OR 28017842[uid]

**PubMed - 2449 in 8/2023 (2447 after deduplication)**

("Liver Cirrhosis"[mesh] OR cirrho*[tw]) AND ("Liver Failure"[mesh] OR decompensat*[tw]) AND ("Predictive Value of Tests"[mesh] OR predict*[tw])

**Embase.com - 4157 in 8/2023 (2741 after deduplication)**

((('Liver Cirrhosis'/exp OR cirrho*:ti,ab) AND (decompensat*:ti,ab)) OR 'decompensated liver cirrhosis'/exp) AND ('predictive value'/exp OR 'predictive validity'/exp OR predict*:ti,ab)

**Scopus.com - 1973 in 8/2023 (121 after deduplication)**

(TITLE-ABS-KEY(cirrho* AND decompensat*)) AND (TITLE-ABS-KEY(predict*))

**Web of Science Core Collection (SCI-EXPANDED, SSCI, A&HCI, CPCI-S, CPCI-SSH, BKCI-S, BKCI-SSH, ESCI, CCR-EXPANDED) - 1788 in 8/2023 (427 after deduplication)**

TS=(cirrho* AND decompensat* AND predict*)

**ClinicalTrials.gov - 97 in 8/2023 (97 after deduplication)**

Condition or Disease Search: Decompensated Cirrhosis

Supplementary Results

**Supplemental Figure 1**: Forest plots demonstrating the individual and pooled hazard ratios per unit increase of MELD in studies stratified by (a) nonviral etiologies of cirrhosis and (b) viral etiologies of cirrhosis.

(a)

(b)

**Supplemental Figure 2:** Forest plots demonstrating the individual and pooled hazard ratios per unit increase (kPa) in LSM by transient elastography in studies stratified by (a) nonviral etiologies of cirrhosis and (b) viral etiologies of cirrhosis.

(a)

(b)

**Supplemental Figure 3**: Forest plot displaying the individual and pooled hazard ratios per unit increase in Child-Turcotte-Pugh Score.


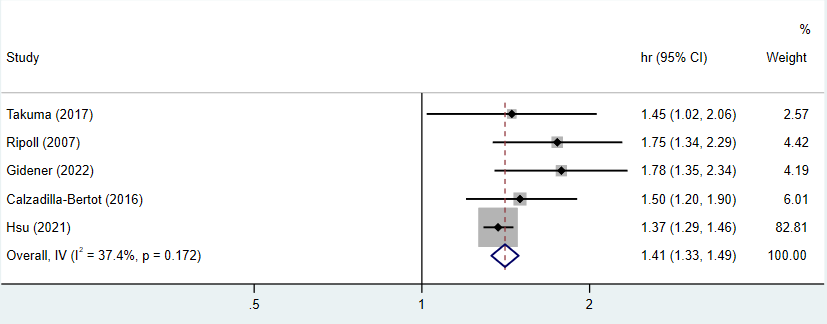


**Supplementary Table S1:** Risk of bias of Studies Using the QUIPS (Quality in Prognosis Studies) Tool.

|  | Risk of Bias (low/moderate/high) | | | | | | |
| --- | --- | --- | --- | --- | --- | --- | --- |
|  | Domain 1: Study Participation | Domain 2: Study Attrition | Domain 3: Prognostic Factor Measurement | Domain 4: Outcome Measurement | Domain 5: Study Confounding | Domain 6: Statistical Analysis and Reporting | Overall Judgment |
| Hu, 1999 | Low | Low | Low | Low | Low | Low | Low |
| Ripoll, 2007 | Low | Low | Low | Moderate | Low | Low | Low |
| Westbrook, 2011 | Moderate | Moderate | Low | Moderate | Moderate | Low | High |
| Berzigotti, Rossi, 2011 | Moderate | Low | Low | Low | Low | Low | Low |
| Berzigotti, Garcia-Tsao, 2011 | Low | Low | Low | Low | Low | Low | Low |
| Robic, 2011 | Low | Low | Low | Moderate | Low | Low | Moderate |
| Kim, 2012 | Low | Low | Low | Low | High | Low | High |
| Procopet, 2014 | Low | Low | Low | Moderate | Low | Low | Low |
| Pérez-Latorre, 2014 | Low | Moderate | Low | Moderate | Low | Low | High |
| Karagozian, 2014 | Low | Moderate | Low | Low | Moderate | Low | Moderate |
| Colecchia, 2014 | Low | Low | Low | Low | Low | Low | Low |
| Wang, 2014 | Low | Moderate | Low | Moderate | Low | Low | Moderate |
| Sultanik, 2016 | Low | Low | Low | Moderate | Low | Low | Moderate |
| Calzadillla-Bertot, 2016 | Low | Low | Low | Low | Low | Low | Low |
| Takuma, 2017 | Low | Low | Low | Low | Low | Low | Low |
| Dillon, 2018 | Low | Low | Low | Low | Moderate | Low | Low |
| Wu, 2018 | Low | Moderate | Low | Moderate | Low | Low | Moderate |
| Guha, 2019 | Low | Low | Low | Low | Low | Low | Low |
| Schwarzer, 2020 | Low | Low | Low | Low | Low | Low | Low |
| Asesio, 2021 | Low | Low | Low | Low | Low | Low | Low |
| Gidener, 2021 | Low | Low | Low | High | Moderate | Low | High |
| Wu, 2021 | Low | Low | Low | Moderate | Low | Low | Moderate |
| Hsu, 2021 | Low | Low | Low | Low | Low | Low | Low |
| Costa, 2021 | Low | Low | Low | Low | Low | Low | Low |
| Calzadilla-Bertot, 2021 | Low | Low | Low | Low | Low | Low | Low |
| Morisco, 2021 | Low | Low | Low | Low | Low | Low | Low |
| Lee, 2021 | Low | Low | Low | Low | High | Low | High |
| Mahmud 2022 | Low | Low | Low | Low | Moderate | Low | Moderate |
| Franzè, 2022 | Low | Low | Low | Low | Low | Low | Low |
| Fujiwara, 2022 | Low | Low | Low | Moderate | Low | Low | Moderate |
| Gidener, 2022 | Low | Low | Low | Low | Moderate | Low | Moderate |
| Jachs, 2022 | Low | Moderate | Low | Low | High | Low | High |
| Liu, “A Novel SAVE,” 2022 | Low | Low | Low | Low | Low | Low | Low |
| Schneider, 2022 | Low | Low | Low | Low | High | Low | High |
| Jindal, 2022 | Low | Low | Low | Low | Low | Low | Low |
| Wong, 2022 | Low | Low | Low | Low | Low | Low | Low |
| Yu, 2022 | Low | Low | Low | Low | Low | Low | Low |
| Karagiannakis, 2023 | Low | Low | Low | Moderate | Moderate | Low | High |
| Kim, 2023 | Low | Low | Low | Low | Low | Low | Low |
| Loomba, 2023 | Low | Low | Low | Moderate | Low | Low | Moderate |
| Marie, 2023 | Low | Low | Low | Low | Low | Low | Low |
| Semmler, 2023 | Low | Low | Low | Low | Low | Low | Low |
| Shearer, 2023 | Low | Low | Low | Low | Low | Low | Low |
| Wong, 2023 | Low | Low | Low | Low | Low | Low | Low |

**Supplementary Table S2** Pooled hazard ratios of supplemental laboratory models.

| Laboratory Marker | Number of studies | Pooled Risk Estimates for All-Cause Decompensation (per unit) |
| --- | --- | --- |
|  |  |  |
| Albumin | 7 | 0.89 (0.86, 0.92) |
| Total Bilirubin | 6 | 1.07 (1.05, 1.10) |
| Platelet Count | 7 | 0.99 (0.98, 0.99) |
| INR | 4 | 1.09 (0.86, 1.39) |
| AST/ALT ratio | 2 | 2.01 (1.44, 2.80) |
